# Perfectionism-related variations in error processing in a task with increased response selection complexity

**DOI:** 10.1017/pen.2022.3

**Published:** 2023-01-11

**Authors:** André Mattes, Markus Mück, Jutta Stahl

**Affiliations:** Department of Individual Differences and Psychological Assessment, University of Cologne, Cologne, Germany

**Keywords:** perfectionism, error negativity, error positivity, cognitive control, action monitoring

## Abstract

Perfectionists strive for a flawless performance because they are intrinsically motivated to set and achieve high goals (personal standards perfectionism; PSP) and/or because they are afraid to be negatively evaluated by others (evaluative concern perfectionism; ECP). We investigated the differential relationships of these perfectionism dimensions with performance, post-response adaptation, error processing (reflected by two components of the event-related potential: error/correct negativity – Ne/c; error/correct positivity – Pe/c) and error detection. In contrast to previous studies, we employed a task with increased response selection complexity providing more room for perfectionistic dispositions to manifest themselves. Although ECP was related to indicators of increased preoccupation with errors, high-EC perfectionists made more errors than low-EC perfectionists. This observation may be explained by insufficient early error processing as indicated by a reduced Ne/c effect and a lack of post-response adaptation. PSP had a moderating effect on the relationship between ECP and early error processing. Our results provide evidence that pure-EC perfectionists may spend many of their cognitive resources on error-related contents and worrying, leaving less capacity for cognitive control and thus producing a structural lack of error processing.

While human beings share basic psychological functions such as information processing, they differ greatly in the specific expression of these functions. One determinant of such variations in psychological functions is perfectionism, that is the stable disposition to strive for flawlessness (Stoeber, 2018).

Perfectionism consists of two key dimensions (Frost, Heimberg, Holt, Mattia, & Neubauer, 1993; Slade & Owens, 1998; Stoeber & Otto, 2006): *Evaluative concern perfectionism* (ECP) describes the tendency to equate mistakes to failure and to be afraid of being evaluated negatively by others based on one’s performance (Frost et al., 1990) and *Personal standards perfectionism* (PSP) is characterised by setting very high goals for one’s own performance and excessively evaluating oneself based on these criteria (Frost, Marten, Lahart, & Rosenblate, 1990). While ECP has been associated with higher levels of neuroticism, external motivation, fear of failure and an avoidance orientation, PSP has been linked to higher levels of conscientiousness, internal motivation, hope of success and an approach orientation (Stricker, Buecker, Schneider, & Preckel, 2019; Stoeber, Damian, & Madigan, 2018). An interactionist approach, the 2×2 model of perfectionism by Gaudreau and Thompson (2010), does not only consider the two main effects of ECP and PSP in isolation but also takes the within-person combination of both perfectionism dimensions into account, allowing to identify four subtypes of perfectionism: pure-EC perfectionism (ECP high, PSP low), pure-PS perfectionism (ECP low, PSP high), mixed perfectionism (ECP high, PSP high) and non-perfectionism (ECP low, PSP low). Note that despite the term “subtype,” the model is based on fully continuous measures of ECP and PSP (Gaudreau, 2013).^1^ Finally, perfectionism also constitutes an important predisposition for psychological disorders (e.g. depression, anxiety and eating disorders; Flett, Hewitt, Blankstein, & Gray, 1998; Flett, Madorsky, Hewitt, & Heisel, 2002; Gnilka, Ashby, & Noble, 2012; Rice & Aldea, 2006; Shafran, Cooper, & Fairburn, 2002) and may even serve as a trans-diagnostic factor (Limburg, Watson, Hagger, & Egan, 2017).

Considering that – independent from the specific perfectionism motivation – perfectionists strive for flawlessness, making an error poses a challenge to perfectionists because it is not compatible with their strivings and concerns. Yet, error processing or – more broadly – response monitoring is an integral part of human behaviour and cognition. It serves to ensure that the intended action is correctly executed and to improve subsequent behaviour if the action is not executed as intended (Wessel, 2018). The error/correct negativity (Ne/c), a component of the event-related potential (ERP), peaks at around 100 ms at fronto-central areas following a response and is usually larger for errors than for correct responses (first mentioned independently by Falkenstein, Hohnsbein, Hoormann, & Blanke, 1991, in a research article; and by Gehring, Coles, Meyer, & Donchin, 1990, in a conference abstract). It is assumed to reflect early error processing, and it is usually independent from error awareness (Wessel, 2012) but may signal the need for more cognitive control (Cavanagh & Frank, 2014; Hester, Foxe, Molholm, Shpaner, & Garavan, 2005) to improve behaviour on the subsequent trial (King, Korb, Cramon, & Ullsperger, 2010; Maier, Yeung, & Steinhauser, 2011; Mattes, Porth, & Stahl, 2022; Ridderinkhof, Ullsperger, Crone, & Nieuwenhuis, 2004). Another error-related ERP component is the error/correct positivity (Pe/c) which peaks at around 300 ms at centro-parietal areas following a response. It has been linked to error awareness (Nieuwenhuis, Ridderinkhof, Blom, Band, & Kok, 2001) and evidence accumulation (Steinhauser & Yeung, 2010, 2012) because it is usually larger for aware errors than for unaware errors or correct responses.

Although response monitoring is a basic psychological function, previous research has shown that perfectionists differ in the extent to which they process errors. For example, PSP has been associated with more error-specific activity in the anterior cingulate cortex (ACC; Barke et al., 2017), the postulated neural generator of the Ne/c (Ridderinkhof et al., 2004). Furthermore, pure-PS and mixed perfectionists were found to yield more intense early error processing in terms of the Ne/c amplitude (Stahl, Acharki, Kresimon, Völler, & Gibbons, 2015). The Ne/c, in turn, has consistently been linked to improved future behaviour (e.g. Debener et al., 2005; Gehring, Goss, Coles, Meyer, & Donchin, 1993; Mattes et al., 2022). Indeed, high-PS perfectionists were shown to exhibit better behavioural adaptation than low-PS perfectionists (i.e. more post-error slowing and a higher post-error accuracy; Barke et al., 2017; Stahl et al., 2015), suggesting that high-PS perfectionists may draw on error processing to optimise their behaviour.

Findings that pure-EC perfectionists show less intense early error processing have previously been interpreted as indicating an attempt to avoid error processing and escape the aversive consequences associated with errors such as negative evaluations by others, being faced with the experience of failure or being confronted with one’s own imperfections (Stahl et al., 2015). Alternatively, in pure-EC perfectionists, an error may not signal the need for cognitive control as much as in the other perfectionism subtypes (Cavanagh & Frank, 2014; Ridderinkhof et al., 2004). While the former explanation assumes a motivational lack of error processing in pure-EC perfectionists, the latter account suggests more of a structural lack of error processing. Findings linking (pure) ECP to increased late error processing as indicated by the Pe/c amplitude (Drizinsky, Zülch, Gibbons, & Stahl, 2016; Tops, Koole, & Wijers, 2013) support the notion of a structural lack of error processing in ECP given that aware error processing or error evidence accumulation seem to work well in EC perfectionists.

Reviewing the current literature on perfectionism and error processing, it is astonishing that almost all studies employed relatively simple two-choice tasks (e.g. Barke et al., 2017; Drizinsky et al., 2016; Pieters et al., 2007; Schrijvers, Bruijn, Destoop, Hulstijn, & Sabbe, 2010; Stahl et al., 2015; Tops et al., 2013), given that perfectionistic tendencies may manifest themselves more clearly in more challenging tasks. Indeed, while meta-analyses suggest that perfectionism is associated with performance in several domains (e.g. academic performance: Madigan, 2019; performance in sports: Hill, Mallinson-Howard, & Jowett, 2018), no perfectionism-related differences in the correct response rate were found in any of the studies referenced above. It seems that overly simplified experimental tasks may not be suitable to investigate perfectionism-related performance differences that are well-established in real-world settings. To avoid such a potential ceiling effect, we designed a more complex yet not unrealistically difficult task that produced enough error trials to reliably assess the error-related components (Ne/c and Pe/c) and triggered perfectionistic traits due to its challenging nature (Stahl et al., 2020). We expected that ECP was associated with less deep processing of errors (avoidance orientation). Regarding the underlying mechanisms of the reduced error processing in ECP, we investigated two competing explanations. It could be that high-EC perfectionists intend to avoid the confrontation with unpleasant consequences of their behaviour, for example errors, and thus avoid error processing altogether (*avoidance hypothesis*). Alternatively, high-EC perfectionists might have fewer cognitive capacities to process their errors because a substantial part of their cognitive capacity is captured by reiterating thoughts about error-related contents or – more generally – worrying (Eysenck, Derakshan, Santos, & Calvo, 2007; Moser, 2017). Hence, error processing might be less deep for high- compared to low-EC perfectionists due to the reduced amount of available cognitive capacity (*capacity hypothesis*). Furthermore, we expected that PSP was associated with a deeper processing of errors (approach orientation). This would allow high-PS perfectionists to draw information from their current behaviour to adapt and optimise their future behaviour (*optimisation hypothesis*). To forward our understanding of error processing in perfectionists in the context of these suggested accounts, we explored a conjoint of behavioural and electrophysiological variables in a task with increased response selection complexity (i.e. eight response options).

## 1. Method

### 1.1. Participants

A total of 95 participants were recruited via e-mail lists and social media groups using a computer-aided registration tool for experiments (CORTEX; Elson & Bente, 2009). They received course credit for participation. Four participants had to be excluded from the analyses because of technical problems with the recording of triggers. One participant was excluded because the correct response rate was not significantly higher than chance suggesting that this participant did not participate conscientiously. The remaining 90 participants (74 female, 15 male, 1 other) had a mean age of 25.08 years (*SD* = 7.58). Post hoc sensitivity analyses showed that this sample size allowed us to detect medium effect sizes of *r* = .30 with an alpha level of 5% and a power of 84% in a multiple regression model with seven predictors (see Statistical analyses for more details). Participants gave their written informed consent, and the study was approved by the ethics committee of the German Psychological Association.

### 1.2. Psychometric assessment

PSP and ECP scores (mean across items) were assessed using the respective *personal standards* (here, Cronbach’s α = .81, range: 1.86–5.71) and *concern over mistakes* scales (here, Cronbach’s α = .91, range: 1.11–5.22) of the German version (Altstötter-Gleich, & Bergemann, 2006) of the Frost Multidimensional Perfectionism Scale (FMPS; Frost et al., 1990), respectively. The items of both perfectionism subscales were measured on a six-point Likert scale ranging from 1 (“trifft gar nicht zu,” roughly: *do not agree at all*) to 6 (“trifft sehr zu,” roughly: *fully agree*). We primarily chose these subscales as measures for PSP and ECP to ensure that our results were comparable to previous studies investigating perfectionism-related variations in error processing (e.g. Barke et al., 2017; Drizinsky et al., 2016; Stahl et al., 2015), all of which used the same subscales. Furthermore, researchers have argued that the other FMPS subscales are not as central to the two higher-order dimensions as personal standards and concern over mistakes (Stoeber, 2018). For example, the parental criticism and expectations subscales are more important in the context of developing perfectionism (Damian, Stoeber, Negru, & Băban, 2013; Rice, Lopez, & Vergara, 2005) and the organisation subscale is often considered as existing in addition to PSP and ECP (Frost et al., 1990; Kim, Chen, MacCann, Karlov, & Kleitman, 2015). Participants completed the questionnaire on a computer and were not able to leave items unanswered.

### 1.3. Procedure and experimental task

**Figure 1.**
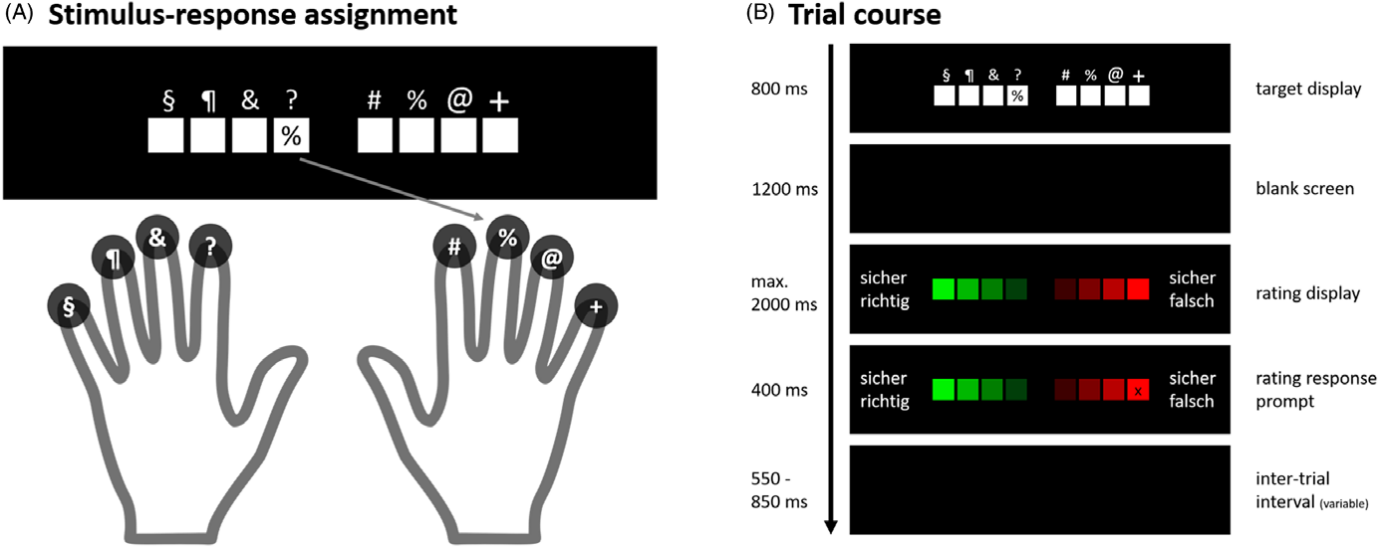
(A) Stimulus-response assignment and (B) trial course. In the example, a percentage sign which is assigned to the right middle finger is the target, so pressing the button with the right middle finger would be the correct response.

We used a task introduced by Stahl et al. (2020) termed the 8ART (eight-alternative response task, Figure 1). Participants had to learn the assignment of eight symbols to eight of their fingers (thumbs excluded). They were instructed to respond as fast and as accurately as possible to the symbol that was presented on the screen by pressing the key with the corresponding finger (Figure 1A). Each of the eight fingers rested on a separate key throughout the experiment. The trial course is illustrated in Figure 1B. At the beginning of each trial, eight white boxes were displayed horizontally representing the eight fingers. Above each box, the corresponding symbol was presented. In one of the boxes, the target symbol was presented and participants had to press the key with the finger that was assigned to the symbol and explicitly not to the location where the target symbol was presented. The target display (800 ms duration) was followed by a blank screen (1200 ms duration). The RT limit was 1200 ms after target onset. On trials on which responses exceeded this limit, the feedback “zu langsam” (“too slow”) was presented and the trial was terminated. On trials with a valid response, the blank screen was followed by a rating display presented for 2000 ms. The eight-point rating scale ranged from “sicher richtig” (“certainly right”) to “sicher falsch” (“certainly wrong”) and could be answered with the eight response keys. To prevent motor response preparation prior to the onset of the rating scale, the scale orientation was assigned randomly on each trial, that is the anchor “certainly right” was randomly presented at the left or right end of the scale. A cross indicated the chosen box for the remaining rating display. The inter-trial interval varied from 550 to 850 ms [for more details on 8ART see, Stahl et al. (2020)].

The participants completed a total of twelve blocks, each consisting of 64 trials so that the eight target symbols appeared at each of the eight locations once in each block. The target symbols were presented in random order. The first block was a practice block and did not contain a response time (RT) criterion, and the target display was visible until a response was given to make sure that participants were able to form the symbol-finger assignment. After each block, participants were given a break of at least two minutes.

After the behavioural task, the participants filled in a questionnaire including the PSP and ECP items (Frost et al., 1990).

### 1.4. Behavioural data

Behavioural data were recorded using eight force-sensitive keys which registered a response when they were pressed with 40 cN or more. A VarioLab AD converter (Becker-Meditec) digitised the analogous signal at a sampling rate of 512 Hz. The actual keys were embedded in individual plastic forms which were separately adjustable for each hand and each finger (not the thumbs). Thus, the hands and fingers had a constant position throughout the experiment. A height adjustable chin rest was placed 85 cm from the screen to reduce general movement artefacts and for a constant eye-screen distance.

### 1.5. Electroencephalography recording and data preprocessing

The EEG was recorded from 63 scalp electrode sites using actiCAP slim/snap (Brain Products), which were arranged according to the standard international 10–20 system (Jasper, 1958) (FP1, FP2, AF7, AF3, AF4, AF8, F7, F5, F3, F1, Fz, F2, F4, F6, F8, FT7, FC5, FC3, FC1, FCz, FC2, FC4, FC6, FT8, T7, C5, C3, C3’, C1, Cz, C2, C4, C4’, C6, T8, TP7, CP5, CP3, CP1, CPz, CP2, CP4, CP6, TP8, P7, P5, P3, P1, Pz, P2, P4, P6, P8, PO7, PO3, POz, PO4, PO8, PO9, O1, Oz, O2, PO10). The active Ag/AgCl electrodes provided by Brain Products were online referenced against the left mastoid electrode. The reference on the left mastoid was an additional electrode (not included in the actiCAP) that was physically connected to the same amplifier as the other scalp electrodes. We placed a second reference electrode (the 64th active electrode from the cap) on the right mastoid that was later used for offline re-referencing (see below). The EEG signal was recorded continuously at a sampling rate of 500 Hz by means of a BrainAmp DC (Brain Products) amplifier. An additional amplifier BrainAmp E×G (Brain Products) was used to record the Electrooculography (EOG) signals using passive Ag/AgCl bipolar electrodes above and below the left eye (vertical eye movements) and on the lower part of the left and right temples (horizontal eye movements).

**Figure 2.**
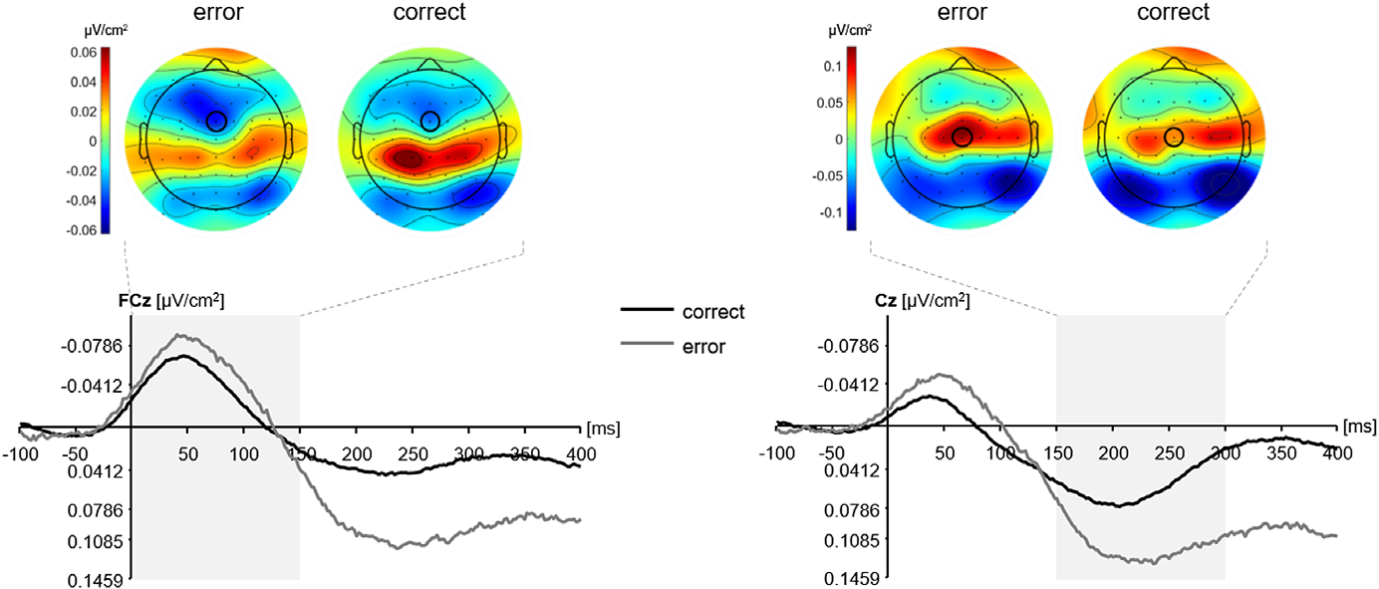
Topographical maps for errors and correct responses in an interval of 0–150 ms following the response (left) and 150–300 ms following the response (right) and grand-average waveforms for errors and correct responses. The error/correct negativity was quantified as the peak amplitude in the area marked grey at FCz (left), and the error/correct positivity was quantified as the peak amplitude in the area marked grey at Cz (right).

The EEG data preprocessing was performed in the EEGLAB toolbox (Delorme & Makeig, 2004) and the ERPLAB plugin (Lopez-Calderon & Luck, 2014) both running under the MATLAB environment (Mathworks). The data were recorded using the BrainVision Recorder (Brain Products) and were imported into EEGLAB using the bva-io plugin version 1.57 (Widmann & Delorme, 2004). The EEG signal was re-referenced offline to the average of the left and right mastoid. A 1 Hz high-pass filter and a 50 Hz notch filter were applied, and a moving window (width: 500 ms; steps: 250 ms) identified segments with a signal exceeding ± 1.000 µV, which were removed. An independent component analysis (ICA) was performed on the continuous EEG signal in the experimental blocks, which was high-pass filtered at 1 Hz. The resulting ICA weights were applied to the continuous EEG signal, which was high-pass filtered at 0.1 Hz. Independent components capturing eye movements (blinks and saccades), muscle activity, or channel noise were removed from the data. Response-locked and baseline corrected epochs (baseline: −100 ms to response onset) starting 100 ms before the response and ending 700 ms after the response in the main task were extracted from the continuous signal and epochs in which participants exceeded the RT criterion were discarded, along with epochs in which the EEG signal exceeded ±150 µV. The epochs were averaged separately for each condition, and a current source density transformation was performed.^2^ The Ne/c peak amplitude was defined as the most negative value at FCz from response onset to 150 ms after the response. Similarly, the Pe/c peak amplitude was defined as the most positive value at the Cz site from 150 to 300 ms after the response. The topographical plots confirmed these locations as the local extrema for the Ne/c and Pe/c (Figure 2), and all conditions met the reliability criterion of at least six trials per participant and condition (Olvet & Hajcak, 2009).

### 1.6. Behavioural variables

The RT was measured as the time difference between the stimulus onset and the point in time at which the first key was pressed with a force of at least 40 cN. Behavioural adaptation was assessed by the pre-post-RT difference RT_pre-post_ and the post-response accuracy. The pre-post-RT difference is a robust estimate of post-error or post-correct slowing and is computed for a given trial *n* as follows (Dutilh et al., 2012):

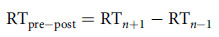




The post-response accuracy was defined as the average correct response rate following correct responses or errors. (For additional exploratory analyses of response force and response certainty, see supplementary material.)

### 1.7. Signal detection

We computed two variables derived from the signal detection theory (Green & Swets, 1966). The bias *c* and sensitivity *d’* parameters are computed as follows:

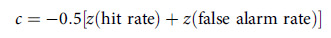




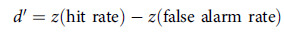

where the function *z*(*p*), *p* ∈ [0,1], is the inverse of the cumulative Gaussian distribution. A “hit” is an error that was correctly identified as such. A “false alarm” is a correct response that was mistakenly evaluated as an error. Higher values in the bias indicate a tendency to evaluate one’s own responses as correct irrespective of the actual response accuracy. Higher values in the sensitivity indicate a better performance in correctly discriminating between correct and erroneous responses. One participant had a false alarm rate of zero; hence, no signal detection parameters could be computed for that participant, reducing the sample size for these analyses by one.

### 1.8. Statistical analyses

We analysed variables derived from signal detection theory, behavioural variables, post-response adaptation and electrophysiological variables.^3^ Signal detection variables and the error rate were analysed in a linear regression model with PSP, ECP, and their interaction term as predictors. The other variables were analysed in a mixed-effects model with PSP, ECP, Response Accuracy (error vs. correct), and all possible interaction terms as predictors.^4^ Participants were treated as random effects, and random intercepts were estimated. Since the RT was measured on the single trial level, random slopes were additionally estimated. Note that the only reason for this procedure was that RTs can relatively easily and reliably be assessed on the single trial level, whereas estimates of electrophysiological variables are commonly derived by averaging the trials to increase the signal-to-noise ratio. Signal detection variables are per definition assessed on the participant level. In all analyses, ECP and PSP were centred and used as continuous predictors. Response type was contrast-coded (correct = −0.5, error = 0.5). Standardised regression coefficients (β) were obtained by repeating the analyses with *z*-standardised variables. In case an interaction reached statistical significance, simple slope analyses were conducted to determine the values of the moderators PSP and ECP for which an effect of Response accuracy was observed. Given that we were primarily interested in error processing (as opposed to unsuccessful error processing, that is error trials that were not signalled as errors), we only included trials in our analyses in which the response accuracy was correctly signalled. This ensured that potentially different mechanisms were not conflated (Stahl et al., 2020). The variance inflation factors for all predictors in all models ranged from 1.04 to 1.54, indicating that multicollinearity was not an issue in our analyses (Eid, Gollwitzer, & Schmitt, 2013, p. 687).

The statistical analyses were conducted in *R* (R Core Team, 2017). The psychometric statistics were computed in the *psych* package (Revelle, 2020). Mixed-effects models were estimated using the *lme4* package (Bates, Mächler, Bolker, & Walker, 2015), and significance tests were conducted using the *lmerTest* package (Kuznetsova, Brockhoff, & Christensen, 2017). To run simple slope analyses on two-way interactions and determine the Johnson-Neyman interval, we resorted to the *interactions* package (Long, 2019). Plots were produced with the *ggplot2* package (Wickham, 2016). The variance inflation factors were computed using the *performance* package (Lüdecke, Ben-Shachar, Patil, Waggoner, & Makowski, 2021).

## 2. Results

Descriptive statistics are presented in Table 1. For the sake of brevity, we only report the statistically significant results in the text. The full analyses including results of predictors that were not statistically significant are displayed in Table 2 (signal detection variables) and Table 3 (behavioural and electrophysiological variables). The data and analyses scripts are available here: https://osf.io/a2jbq/

**Table 1.** Descriptive statistics

Variable	Correct		Error		Total	
	*M*	*SD*	*M*	*SD*	*M*	*SD*
Personal standards perfectionism					4.23	0.79
Evaluative concern perfectionism					2.98	1.05
Error rate [%]					21.88	13.69
Bias					1.59	0.33
Sensitivity					0.98	0.35
Response time [ms]	772	47	794	67	773	44
Pre-Post-RT difference [ms]	0	32	22	40	1	25
Post-response accuracy [%]^a^	78.20	11.38	78.27	15.66	77.73	12.70
Error/correct negativity [µV/cm^2^]	−0.09	0.08	−0.13	0.11	−0.10	0.08
Error/correct positivity [µV/cm^2^]	0.11	0.15	0.19	0.16	0.12	0.15

Mean (M) and standard deviation (SD) for all relevant variables split by response accuracy (error vs. correct).

a The mean of the total post-response accuracy is smaller than the means of the post-response accuracy for errors and correct responses. Nevertheless, for each individual participant, the total post-response accuracy lies between the post-response accuracy for errors and correct responses. See Kievit, Frankenhuis, Waldorp and Borsboom (2013) on how patterns on the group level may differ from patterns on the participant level.

**Table 2.** Results of the regression analyses for the signal detection and electrophysiological variables

Predictor	*B*	*SE*	β	*p*
*Error rate*				
(Intercept)	0.23	0.02	0.07	<.001
PSP	−0.04	0.02	−0.24	.056
ECP	0.05	0.02	0.42	.001
PSP × ECP	−0.02	0.02	−0.12	.194
*Bias*				
(Intercept)	1.57	0.04	−0.05	<.001
PSP	0.07	0.05	0.18	.167
ECP	−0.11	0.04	−0.36	.004
PSP × ECP	0.04	0.04	0.10	.287
*Sensitivity*				
(Intercept)	1.01	0.04	0.07	<.001
PSP	−0.05	0.06	−0.11	.386
ECP	0.08	0.04	0.23	.077
PSP × ECP	−0.06	0.04	−0.14	.157

Regression coefficient (b), standard error (SE), standardised regression coefficient (β), and p-value (p) for the predictors in each model.

**Table 3.** Results of the mixed-effects model analyses for behavioural and electrophysiological variables

Predictor	*B*	*SE*	β	*p*
*Response time*				
(Intercept)	785.65	5.60	0.10	<.001
Accuracy	22.16	7.87	0.15	.006
PSP	−2.23	7.94	−0.01	.779
ECP	−2.11	5.76	−0.01	.715
Accuracy × PSP	−1.88	11.15	−0.01	.866
Accuracy × ECP	−5.10	8.07	−0.04	.529
PSP × ECP	−6.63	5.63	−0.04	.242
Accuracy × PSP × ECP	0.70	7.95	<0.01	.930
*Post-response time*				
(Intercept)	12.56	3.15	0.04	<.001
Accuracy	24.6	5.50	0.65	<.001
PSP	−6.52	4.46	−0.14	.147
ECP	1.27	3.24	0.04	.696
Accuracy × PSP	−9.71	7.78	−0.20	.216
Accuracy × ECP	−11.88	5.66	−0.33	.039
PSP × ECP	−3.22	3.15	−0.07	.310
Accuracy × PSP × ECP	−5.88	5.50	−0.13	.288
*Post-response accuracy*				
(Intercept)	0.77	0.01	−0.08	<.001
Accuracy	>−0.01	0.01	−0.01	.904
PSP	0.05	0.02	0.27	.028
ECP	−0.06	0.02	−0.42	<.001
Accuracy × PSP	0.02	0.01	0.11	.127
Accuracy × ECP	−0.02	0.01	−0.13	.065
PSP × ECP	0.02	0.01	0.14	.117
Accuracy × PSP × ECP	<0.01	0.01	0.02	.644
*Error/correct negativity*				
(Intercept)	−0.11	0.01	0.03	<.001
Accuracy	−0.03	0.01	−0.34	<.001
PSP	0.02	0.01	0.13	.249
ECP	0.01	0.01	0.14	.230
Accuracy × PSP	>−0.01	0.01	−0.01	.910
Accuracy × ECP	0.01	0.01	0.14	.160
PSP × ECP	−0.01	0.01	−0.06	.506
Accuracy × PSP × ECP	−0.02	0.01	−0.15	.043
*Error/correct positivity*				
(Intercept)	0.15	0.02	−0.01	<.001
Accuracy	0.08	0.01	0.50	<.001
PSP	−0.04	0.02	−0.20	.097
ECP	−0.02	0.02	−0.10	.366
Accuracy × PSP	>−0.01	0.02	−0.01	.940
Accuracy × ECP	−0.02	0.01	−0.11	.208
PSP × ECP	< 0.01	0.02	0.02	.817
Accuracy × PSP × ECP	>−0.01	0.01	−0.01	.917

Regression coefficient (b), standard error (SE), standardised regression coefficient (β), and p-value (p) for the predictors in each model.

### 2.1. Signal detection variables

**Figure 3.**
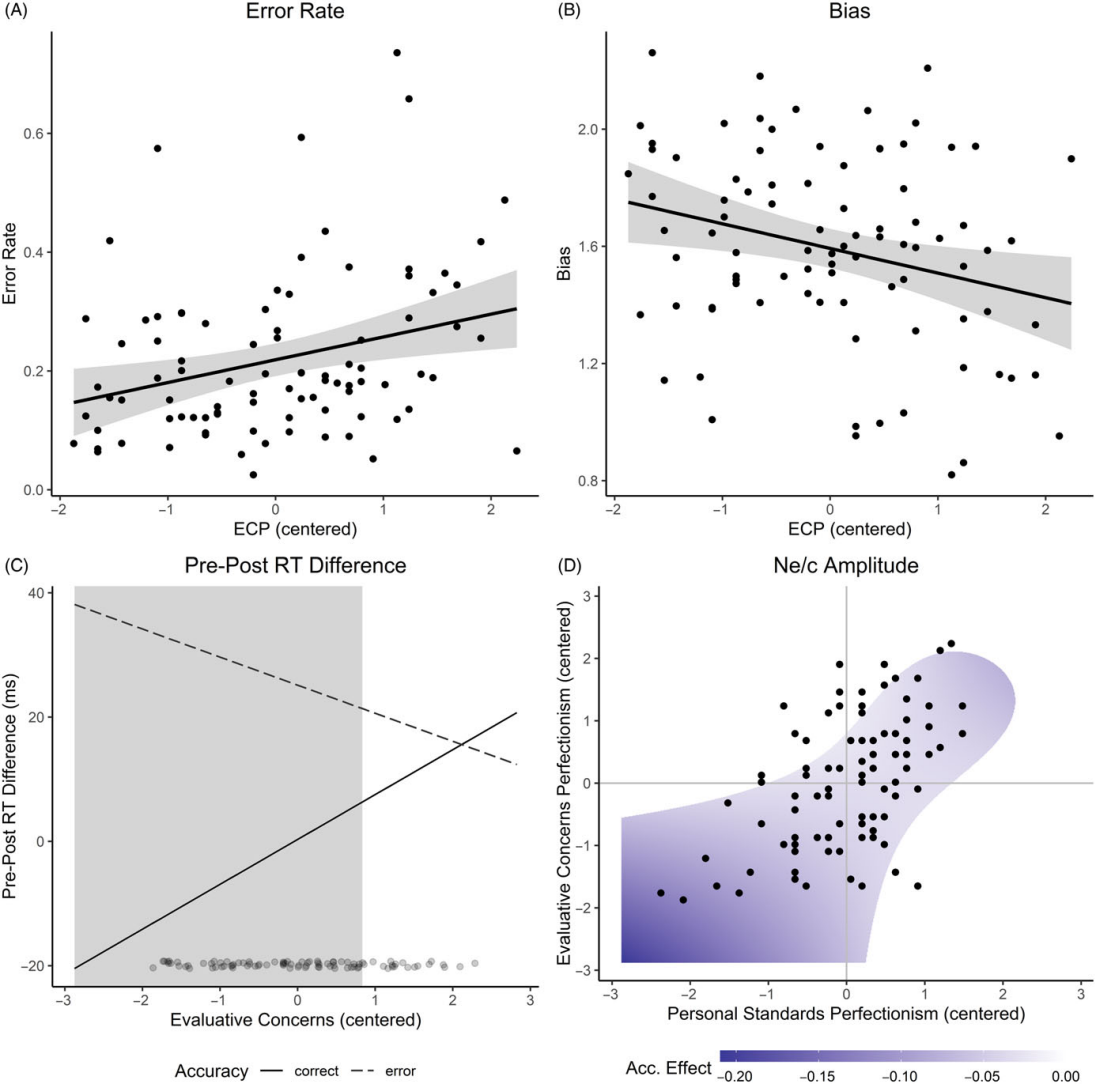
Results regarding perfectionism dimensions. The dots indicate the observed values of PSP and ECP. The grey area in panels A and B represents the 95% confidence interval around the regression line. The grey area in panel C indicates the range of the moderator depicted on the *x*-axis for which the slope of Response Accuracy is significant. The colours in panel D indicate the value of the simple slope for Response Accuracy (“Acc. Effect”) for each combination of PSP and ECP. White represents insignificant slopes. More information on how panel D was generated is provided in the supplementary material.

The regression model predicting the error rate by ECP, PSP, and their interaction term yielded a significant effect of ECP, *b ± SE* = 0.05 ± 0.02, *t*(86) = 3.48, *p* = .001. Higher levels of ECP were associated with higher error rates (Figure 3A).

For the bias, a significant ECP effect emerged, *b* = 0.11 ± 0.04, *t*(85) = 2.93, *p* = .004. The higher ECP was, the more participants tended to evaluate their responses as errors, independent from the actual response accuracy (Figure 3B).

### 2.2. Behavioural variables and post-response adaptation

The mixed-effects model for RT only yielded a significant main effect for response accuracy, *b* = 22.16 ± 7.87, *t*(81.56) = 2.82, *p* = .006. Errors (*M* ± *SE*: 794 ± 7.08 ms) were significantly slower than correct responses (772 ± 4.95 ms).

For the pre-post-RT difference, that is the more robust estimate of changes in post-RT, we found a significant effect of response accuracy, *b* = 24.60 ± 5.50, *t*(86) = 4.47, *p* < .001, and a response accuracy by ECP interaction, *b* = −11.88 ± 5.66, *t*(86) = −2.10, *p* = .039. The pre-post-RT difference was larger for errors (22.12 ± 4.24 ms) than correct responses (0.14 ± 3.37 ms), indicating more slowing following errors than following correct responses. However, this observation was restricted to participants with low- to-medium scores of ECP as indicated by the Johnson-Neyman interval [0.83, 35.81] (Figure 3C). The difference between errors and correct responses was only significant for ECP values outside this interval.

The regression model predicting post-response accuracy yielded a significant effect for PSP, *b* = 0.05 ± 0.02, *t*(86) = 2.23, *p* = .028 and a significant effect for ECP, *b* = −0.06 ± 0.02, *t*(86) = −3.69, *p* < .001. The higher the PSP was, the higher the probability of giving a correct response on the following trial was. For ECP, the relationship was reversed. Increasing ECP scores were associated with a decreasing probability of a correct response on the subsequent trial. Note, however, that both main effects may be explained by a generally higher response accuracy for PS perfectionists and a generally lower response accuracy for EC perfectionists.

### 2.3. Electrophysiological variables

**Figure 4.**
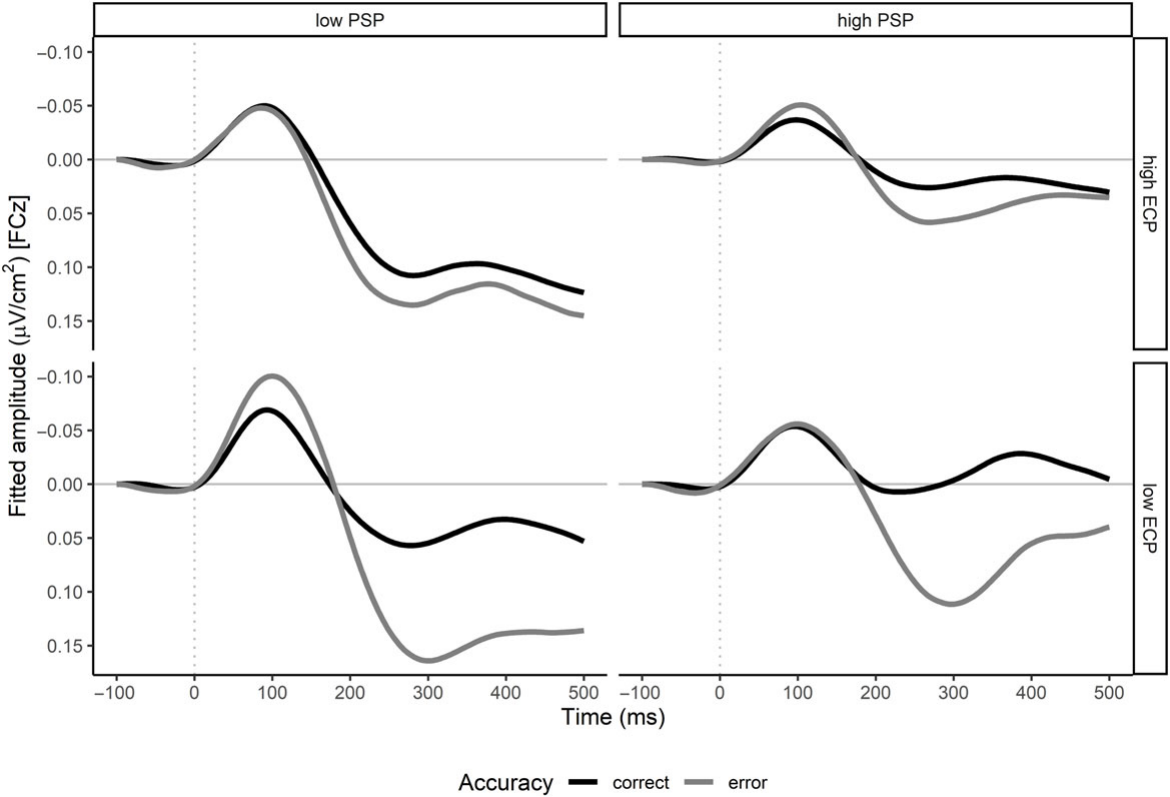
Fitted grand-average waveforms (derived from mixed-effects models) at FCz for errors and correct responses for pure-EC perfectionists (upper left), mixed perfectionists (upper right), non-perfectionists (lower left), and pure-PS perfectionists (lower right). For illustrative purposes, the waveforms were low-pass filtered at 5 Hz. To obtain the waveforms, a separate mixed-effects model with response accuracy, PSP, ECP, and all possible interactions was computed for each time step. Next, each mixed-effects model was used to predict the amplitude for all possible combinations of response accuracy (error and correct), high PSP or ECP (i.e. mean plus one standard deviation) and low PSP or ECP (mean minus one standard deviation), resulting in the presented waveforms. For more details on the computation of the waveforms and for the corresponding waveforms at the Cz electrode site, see supplementary material.

The mixed-effects model for the Ne/c amplitude yielded a significant Response Accuracy main effect, *b* = −0.03 ± 0.01, *t*(86) = 3.83, *p* < .001, and a response accuracy by PSP-by-ECP interaction effect, *b* = 0.02 ± 0.01, *t*(86) = 2.05, *p* = .043. On average, the Ne/c amplitude was more pronounced after errors (0.13 ± 0.01 µV/cm^2^) than after correct responses (0.09 ± 0.01 µV/cm^2^). The results of a simple slope analysis to further investigate the three-way interaction are illustrated in Figure 3D. The difference in Ne/c amplitude between errors and correct responses was largest for non-perfectionists and mixed-perfectionists, pointing towards more error-specific activity in these perfectionism subtypes. A striking observation is that for pure-EC perfectionists, there was hardly a difference in the Ne/c amplitude, suggesting a lack of error-specific activity. Fitted waveforms for the different perfectionism subtypes are presented in Figure 4.

For the Pe/c amplitude, we only found a significant response accuracy effect, *b* = 0.08 ± 0.01, *t*(86) = 6.32, *p* < .001. Errors showed a higher Pe/c amplitude than correct responses (0.19 ± 0.02 µV/cm^2^ and 0.11 ± 0.02 µV/cm^2^, respectively). There were no perfectionism-related effects regarding the Pe/c amplitude. This is surprising because the fitted waveform in Figure 4 suggests that the Pe/c amplitude covaries with the two perfectionism dimensions. However, our analyses are based on the Pe/c amplitude at the electrode site Cz while Figure 4 displays the (fitted) waveforms at the electrode site FCz. For exploratory purposes, we repeated the analyses for the Pe/c amplitude measured at FCz. Note that although most scientific studies use the EEG signal at the Cz electrode to quantify the Pe/c, a notable body of literature investigates the Pe/c at the FCz site (e.g. Endrass, Reuter, & Kathmann, 2007; Morris, Yee, & Nuechterlein, 2006). The mixed-effects model yielded a significant main effect for response accuracy, *b* = 0.09 ± 0.01, *t*(86) = 7.43, *p* < .001, and PSP, *b* = −0.04 ± 0.02, *t*(86) = −2.00, *p* = .049, and a significant response accuracy by ECP interaction effect, *b* = −0.04 ± 0.01, *t*(86) = −3.22, *p* = .002. Errors showed a higher Pe/c amplitude than correct responses (0.17 ± 0.02 µV/cm^2^ and 0.08 ± 0.01 µV/cm^2^, respectively). However, this difference was only significant for participants with low- to-medium scores of ECP as indicated by the Johnson-Neyman interval [1.24, 5.97]. For ECP values outside this interval, the difference in Pe/c amplitude between correct responses and errors was significant. Furthermore, the higher PSP was, the smaller was the Pe/c amplitude regardless of whether the response was correct or erroneous.

In the supplementary material, we report additional analyses on the non-transformed (i.e. not CSD-transformed) data and analyses including the error rate and age as covariates.

## 3. Discussion

In the framework of the 2×2 model of perfectionism (Gaudreau & Thompson, 2010), we investigated error processing in a task with increased response selection complexity. The variables of interest captured task performance, post-response adaptation, error detection and electrophysiological error processing indicators, which demonstrated differential processing of errors among within-person combinations of ECP and PSP.

Higher levels of ECP were associated with a higher error rate than lower levels of ECP, which indicates that high ECP is linked to worse task performance. Although ECP has been linked to worse performance in several domains such as worse academic performance (Madigan, 2019) or reduced goal achievement at the work place (Ocampo, Wang, Kiazad, Restubog, & Ashkanasy, 2020), no relationship with the error rate has been found in experimental tasks so far (e.g. Schrijvers et al., 2010; Stahl et al., 2015; Tops et al., 2013). Unlike previous studies, we employed a task with a more complex response selection to avoid a potential ceiling effect in performance due to the simplicity of the task. It seems that the increased complexity of our task facilitated the detection of perfectionism-related differences in task performance. Further analyses provide several potential reasons for the observed relationship between ECP and the error rate. First, ECP was associated with less post-error slowing, a phenomenon that is linked to post-response adaptation (Laming, 1968; Rabbitt, 1966). Post-error slowing has been hypothesised to emerge from an increased response caution following errors, allowing participants to collect more information before the response is initiated on the subsequent trial, hence preventing future errors. Other researchers have suggested that post-error slowing might reflect an orienting response aimed at identifying the source of the error (Houtman & Notebaert, 2013; Notebaert et al., 2009), which would also allow participants to avoid committing repeated errors (Wessel, 2018). Regardless of the specific mechanism of post-error slowing, it seems that high-EC perfectionists do not implement this post-trial correction mechanism as much as low-EC perfectionists, which could explain their poorer task performance. Second, early error-specific activity as indicated by the difference between the Ne and Nc peak amplitude was low for pure-EC perfectionists. In fact, for high levels of pure-ECP, the regression model predicted no error-specific activity at all, while mixed perfectionists and pure-PS perfectionists did display error-specific activity (Figure 3D). It seems that pure-EC perfectionists process errors less deeply and more similarly to correct responses compared to other perfectionism subtypes and may even lack error-specific processing altogether (Stahl et al., 2015). Perhaps the combination of less error-specific activation in the early stages of error processing and less post-trial correction may have led to the poorer performance that was observable for high ECP.

We expected that high-PS perfectionists compared to low-PS perfectionists processed errors more deeply to optimise their behaviour. On a descriptive level, we found that high-PS perfectionists tended to perform better than low-PS perfectionists in terms of response accuracy. However, this effect was not statistically significant. Furthermore, we found that PSP moderated the relationship between ECP and early response monitoring as indicated by the Ne/c amplitude (for further discussion, see below). It is surprising that we did not find convincing evidence for the optimisation hypothesis of PSP considering numerous reports in the literature that highlight the adaptive aspect of PSP in response to errors. For instance, Stahl et al. (2015) found an increased post-error accuracy for high-PS perfectionists. Similarly, Barke et al. (2017) reported that PSP was associated with increased activity in brain areas that are related to response monitoring (ACC; Ridderinkhof et al., 2004) and goal orientation and post-response adaptation (putamen; Hester et al. 2009; Linke et al., 2010). Taken together, these findings suggest that high-PS perfectionists process errors more deeply allowing them to draw more information from their errors and consequently successfully adapt their subsequent behaviour. Our study could not contribute to this body of evidence.

**Figure 5.**
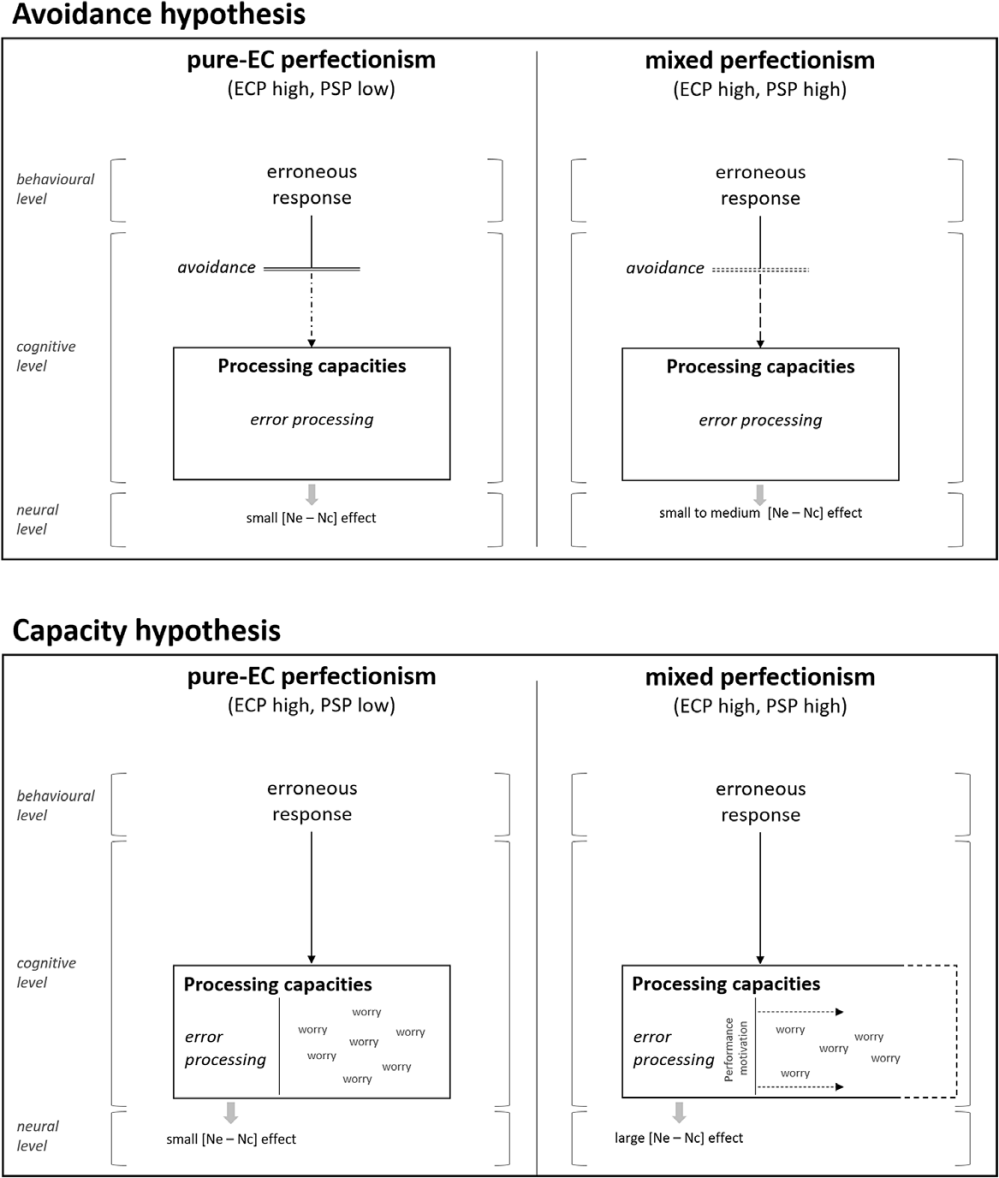
Illustration of the avoidance hypothesis (upper panel) and the capacity hypothesis (lower panel). The avoidance hypothesis postulates that when ECP is high and personal standards perfectionism (PSP) is low (i.e. pure-EC perfectionists; upper left panel), error processing is avoided altogether. When PSP is high (i.e. mixed perfectionists; upper right panel), error processing might be attenuated or not impacted at all. The capacity hypothesis claims that when ECP is high and PSP is low (lower left panel), pure-EC perfectionists dispose of less capacities that could be allocated to error processing because parts of the cognitive capacities are captured by worrying. Furthermore, they are not intrinsically motivated to perform well. Hence, error processing is diminished. When ECP is high and PSP is also high (lower right panel), mixed perfectionists also dispose of less capacities. However, because they are intrinsically motivated to perform well, they reallocate resources to error processing in a compensatory effort, resulting in intensive error processing.

Although our results regarding the optimisation hypothesis of PSP were not as convincing as in previous studies, we found clear evidence in favour of our hypothesis that ECP was associated with less deep error processing. However, it is still unclear whether this is due to avoidance of error processing or capacity limitations. Figure 5 illustrates both accounts and their predictions regarding the Ne/c amplitude. The *avoidance hypothesis* postulates that high-EC perfectionists avoid error processing because of the unpleasant consequences associated with errors. Hence, pure-EC perfectionists should show less error-specific activity in terms of the Ne/c amplitude than non-perfectionists. For both perfectionism subtypes, PSP is low. When PSP is high, the associated intrinsic motivation to perform flawlessly (Nordin-Bates, Raedeke, & Madigan, 2017) might overwrite the fear of negative evaluation to a certain extent. Hence, error-specific activity in mixed perfectionists should be higher than in pure-EC perfectionists, but lower than in pure-PS perfectionists because pure-PS perfectionists have a high intrinsic motivation and no fear of negative evaluation. The *capacity hypothesis* postulates that high-EC perfectionists have a limited cognitive capacity because much of their capacity is captured by error-related contents or – more generally – worrying (Moser, Moran, Schroder, Donnellan, & Yeung, 2013; Moser, 2017). As a consequence, pure-EC perfectionists process errors less deeply than non-perfectionists, resulting in less error-specific activity in terms of the Ne/c amplitude. Furthermore, the account predicts that because of their high internal motivation, mixed perfectionists may be able to allocate additional resources to error processing, compensating for the reduced capacities. This compensatory effort in mixed perfectionists may produce more error-specific activity than both in pure-EC perfectionists (who lack internal motivation) and in pure-PS perfectionists (who have sufficient processing capacities and thus do not show a compensatory effort) because greater resources need to be allocated to error processing to make up for the capacities that are captured by worrying (Moser, 2017). To sum up, both accounts converge in their predictions regarding the pattern of error-specific activity for pure-EC perfectionists. However, they differ in their predictions for mixed perfectionists (intensity of error processing for the avoidance hypothesis: pure-PSP > mixed perfectionism > pure-ECP; for the capacity hypothesis: mixed perfectionism > pure-PSP > pure-ECP). Hence, the comparison of the error-specific activity pattern between mixed and pure-PS perfectionists allows to draw conclusions regarding the underlying mechanism of ECP-related error processing.

As Figure 3D shows, our results regarding the Ne/c amplitude support the capacity hypothesis. The difference in the Ne/c amplitude between errors and correct responses was larger for mixed perfectionists than for pure-PS perfectionists. Furthermore, for pure-EC perfectionists, this accuracy effect was very small or even insignificant. This pattern replicates findings by Stahl et al. (2015). Apart from the Ne/c amplitude results, our data provide even more evidence in favour of the capacity hypothesis. ECP was associated with a bias to declare the given response as an error. Note that despite this bias, ECP was not associated with a more accurate classification of the given response. Hence, the declaration of the response as an error was independent of the actual response accuracy and might reflect a preoccupation of high-EC perfectionists with errors. This preoccupation may be a marker of the limited cognitive capacities due to error-related contents and worrying as suggested by the capacity hypothesis (Figure 5). The avoidance hypothesis, however, would not predict a preoccupation with errors of EC perfections because they should avoid error processing altogether. Our data demonstrate that EC perfectionists are very much concerned with errors. Finally, we found that the difference in the Pe/c amplitude between errors and correct responses at the FCz electrode site was restricted to low- to-medium levels of ECP and disappeared for high levels of ECP. The Pe/c has been associated with error awareness (Nieuwenhuis et al., 2001) and error evidence accumulation (Steinhauser & Yeung, 2010, 2012). The fact that – for high levels of ECP – the Pe/c peaked just as high for correct responses as for errors (Figure 4) may indicate that high-EC perfectionists tended to interpret correct responses as errors (or accumulate evidence for errors when responses were actually correct). This electrophysiological mechanism may have translated to a bias in high-EC perfectionists to declare correct responses as errors on the behavioural level.

### 3.1. Limitations and future research

Although our data lend more support for the capacity hypothesis of ECP than for the avoidance hypothesis, our study is not suitable to falsify the avoidance hypothesis. The primary purpose of our study was to explore perfectionism-related variations in error processing in a task with increased response selection complexity. While we were able to interpret our findings in the context of the avoidance, capacity, and optimisation hypotheses, these interpretations were post hoc. Furthermore, we did not employ an adjustment for multiple comparisons due to the exploratory nature of our study. Individual effects should thus be interpreted with caution. While our sample size was large enough to detect medium effects with an adequate probability, small effects may not have been detected. Our study provides standardised effect sizes that may serve as a basis for a priori sample size calculations in future studies. Most importantly, however, future studies could be designed more specifically to test either one of the hypotheses or reconcile them in an overarching account. For example, future studies may employ the presence vs. absence of a detection rating to specifically test the avoidance hypothesis: If high-EC perfectionists avoid error processing, they should do so when no detection rating is required, but they should fail to do so when they are forced to rate the accuracy of their responses (Drizinsky et al., 2016).

It should further be noted that in our study, errors were slower than correct responses, replicating findings by Stahl et al. (2020) and Porth, Mattes and Stahl (2022) who used the same experimental task in different samples. Other studies using a simpler task reported faster errors compared to correct responses (e.g. Stahl et al., 2015). This observation suggests that some of the errors in our task may originate from a different mechanism than the errors in previous studies. For example, fast errors are often assumed to be the result of an impulsive, premature response (Novikov et al., 2017). Slow errors may emerge on trials on which the correct response is not identified but an arbitrary response is initiated nonetheless to avoid exceeding the time limit (Murphy, Boonstra, & Nieuwenhuis, 2016; Stahl et al., 2020). This mechanism is more likely to be relevant in tasks with an increased response selection complexity like in our study (Stahl et al., 2020). We cannot rule out that our findings are restricted to this type of error, although Stahl et al. (2020) showed that this task provokes both fast and slow error types. Future studies may contrast a simpler and a more complex task within-participants to explore whether these potential qualitative differences in errors are linked to different dimensions/subtypes of perfectionism.

Finally, a recent debate has called into question whether variables capturing individual differences are better explained by a small set of basic personality traits like the Big Five (Bainbridge et al., 2022). While PSP and ECP are related to conscientiousness and neuroticism, respectively (Stoeber et al., 2018; Stricker et al., 2019), researchers have stressed that the concepts are qualitatively different. Furthermore, perfectionism has been shown to play an important conceptual role in a series of psychological disorders (Limburg et al., 2017), perhaps above and beyond the Big Five. Hence, studying perfectionism-related variations in cognitive mechanisms has a large potential to contribute to understanding these disorders better and developing adequate treatments for them.

## 4. Conclusion

Employing a more challenging task compared to simpler tasks has proven helpful in exploring differential mechanisms of response monitoring associated with within-subject PSP-by-ECP combinations. Future studies on perfectionism-related variations in error processing may benefit from increasing the response selection complexity of their experimental tasks to avoid potential ceiling effects. Our study contributes to developing a testable model of error processing in perfectionists.

## Footnotes

1 The term “subtype” is merely used to describe more easily the location of a person on the two-dimensional plane that results from the within-person combinations of ECP and PSP.

2 Analyses of the non-transformed data are presented in the supplementary material.

3 Results of the analyses of response force and response certainty are provided in the supplementary material.

4 Additional analyses including the error rate and age as covariates are presented in the supplementary material.

## Supplementary material

For supplementary material accompanying this paper visit https://doi.org/10.1017/pen.2022.3. click here to view supplementary material
