# Supplementary material for: Perfectionism-related variations in error processing in a task with increased response selection complexity
Source: Personal Neurosci. 2023 Jan 11;5:e12. doi: 10.1017/pen.2022.3 (PMC9880962; doi:10.1017/pen.2022.3)
Supplement: Supplementary file 1 [file S2513988622000037sup001.pdf]

Perfectionism-related variations in error processing in a task with increased response selection complexity

André Mattes, Markus Mück, Jutta Stahl

Department of Individual Differences and Psychological Assessment, University of Cologne

Author Note

This research was supported by the German Research Foundation (STA 1035/7-1) awarded to Jutta Stahl.

Correspondence concerning this article should be addressed to André Mattes, Department of Individual Differences and Psychological Assessment, University of Cologne, Pohligstraße 1, 50969 Köln, Germany. E-Mail: andre.mattes@uni-koeln.de

Supplementary Material

Non-transformed ERP data

For our main ERP analyses, we applied a CSD transformation to the data because it reduces overlapping activity from different electrode sites (Kamarajan et al., 2015) and thus, confounding effects of other neurocognitive processes (e.g. overlap of stimulus-locked P3 with the response-locked Pe; Stahl et al., 2020, supplement). Furthermore, CSD transformation sharpens the location and intensity of the current generators (Kayser & Tenke, 2006; Tenke & Kayser, 2005). We quantified the CSD-transformed Pe/c as the most positive peak at the Cz electrode in the time window from 150 to 300 ms following the response because it was maximal at this electrode site in this time window (see also Figure 2 in the main text). This electrode site and time window suggest that our main ERP analyses were conducted on the so-called “early” Pe/c. Another Pe/c described in the literature is the “late” Pe/c which has a more parietal distribution and a later peak (Arbel & Donchin, 2009; Ruchsow et al., 2005; van Veen & Carter, 2002). To ensure that the CSD transformation did not attenuate or even remove the late Pe/c, we inspected the waveforms at the midline electrodes in the CSD-transformed data and compared them to the waveforms in the non-CSD-transformed data.

Supplementary Figure 1 highlights two important findings: First, the (early) Pe/c emerged more clearly in the CSD-transformed data than in the non-CSD-transformed data, supporting the choice to apply a CSD transformation to the data. Second, a more parietal, late Pe/c was not observable, neither in the CSD-transformed nor in the non-CSD-transformed data. Both findings are supported by an inspection of the topographical maps in Supplementary Figure 2. The positive activation is most pronounced at frontal and central electrodes in the non-CSD-transformed data. The topographical maps of the CSD-transformed data demonstrate that the positive activation can be most clearly observed at the Cz electrode site.

Supplementary Figure 1

Response-locked ERPs in non-CSD-transformed and CSD-transformed data

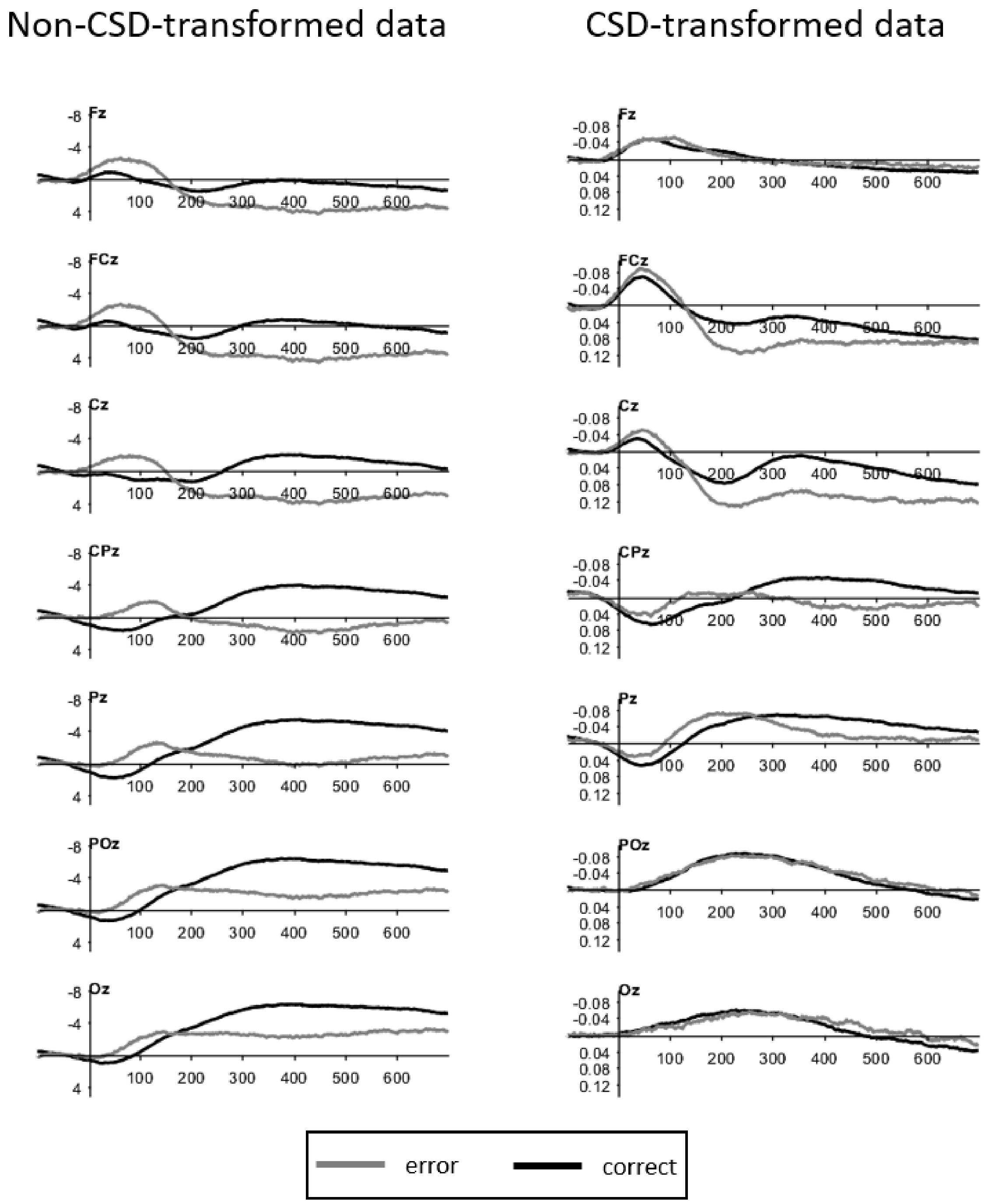

Note. The left panels show the response-locked ERPs at the midline electrodes for errors (black line) and correct responses (grey line) in the non-CSD-transformed data (in µV). The right panels show the corresponding ERPs after CSD-transformation (in µV/cm^2).

Supplementary Figure 2

Topographical maps for non-CSD-transformed and CSD-transformed data

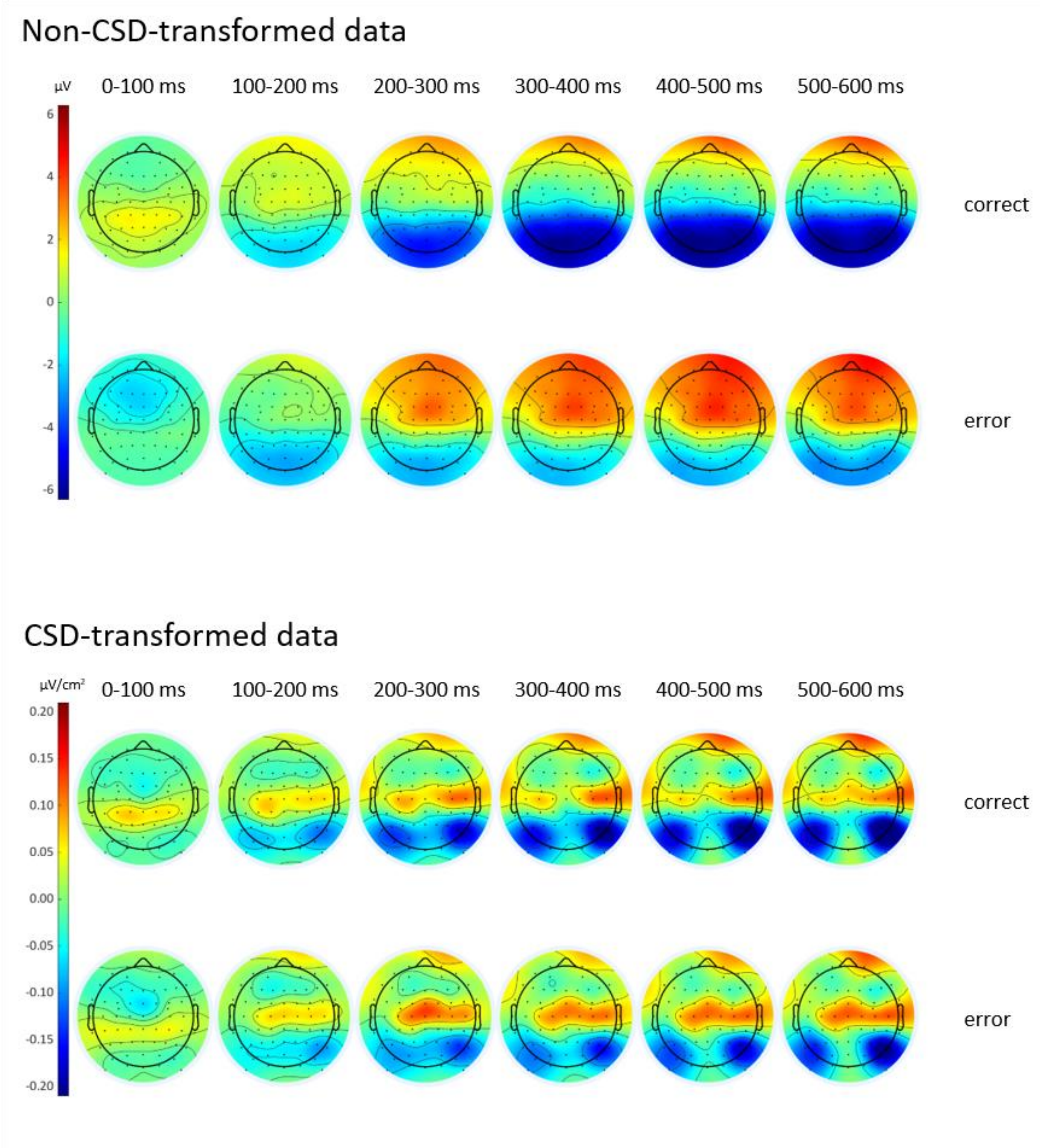

Note. The topographical maps display the average distribution of activation in 100 ms time intervals following response onset.

Although these findings suggest that the CSD-transformed data is more apt to quantify the Ne/c and Pe/c, we repeated the ERP analyses for the non-CSD-transformed data. These analyses may be useful to compare patterns in our data with results reported in the literature based on non-CSD-transformed data. The results are displayed in Supplementary Table 1.

Supplementary Table 1

Regression coefficient (b), standard error (SE), standardized regression coefficient (β), and p-value (p) for the predictors in the mixed models of the non-CSD-transformed data

Predictor	Error Negativity [FCz]				Error Positivity [Cz]				Error Positivity [FCz]			
	b	***SE***	**β**	***p***	***b***	***SE***	**β**	***p***	***b***	***SE***	**β**	***p***
(Intercept)	-3.05	0.25	0.05	<.001	4.05	0.40	0.03	<.001	4.68	0.49	0.05	<.001
Accuracy	-3.12	0.35	-1.02	<.001	2.89	0.49	0.66	<.001	3.12	0.49	0.62	<.001
PSP	0.01	0.36	< 0.01	.973	-1.33	0.57	-0.24	.022	-1.45	0.70	-0.23	.041
ECP	0.46	0.26	0.16	.082	0.05	0.41	0.01	.907	0.21	0.51	0.04	.685
Accuracy x PSP	0.21	0.50	0.05	.679	0.97	0.70	0.18	.167	0.89	0.69	0.14	.199
Accuracy x ECP	0.44	0.37	0.15	.233	-1.18	0.51	-0.28	.022	-1.35	0.50	-0.28	.008
PSP x ECP	-0.34	0.25	-0.05	.181	-0.32	0.40	-0.06	.431	-0.60	0.49	-0.10	.224
Accuracy x PSP x ECP	0.13	0.35	0.04	.710	0.67	0.49	0.13	.177	0.66	0.49	0.11	.181

Controlling for error rate

It has been shown that the Ne amplitude is related to the number of errors made by the participant: the more errors, the smaller the Ne amplitude (Fischer et al., 2017). Furthermore, there is evidence that the Ne is only larger than the Nc when correct response are more frequent than errors. When the frequency distribution was reversed (i.e. more errors than correct responses), no Ne-Nc peak difference was found because the Ne was (descriptively) diminished and the Nc was enhanced (Núñez Castellar et al., 2010). Hence, the Ne-Nc peak difference is at least partially a function of the error rate. Since we have found effects of perfectionism on error rate, it is crucial to ensure that perfectionism-related variations in the Ne/c are not due to different error rates. To this end, we reran the ERP analyses including error rate as an additional predictor and thus controlling for the impact of error rate. The results are presented in Supplementary Table 2. The pattern of results was the same as for the analyses reported in the main text.

Controlling for age

A widely investigated individual difference in cognitive functioning and error processing is age (e.g. Niessen et al., 2017; Overhoff et al., 2021). To ensure that our results were not biased by this variable, we repeated all of our analyses controlling for age. We found a positive relationship between age and the error rate, and age and response times. In other words, older participants made more errors and responded more slowly than younger participants. Apart from these findings, the pattern of results was the same as in the main analyses. All results are displayed in Supplementary Table 3, Supplementary Table 4, and Supplementary Table 5.

Supplementary Table 2

Regression coefficient (b), standard error (SE), standardized regression coefficient (β), and p-value (p) for the predictors in the mixed models on CSD-transformed data controlling for error rate

Predictor	Error Negativity [FCz]				Error Positivity [Cz]				Error Positivity [FCz]			
	b	*SE*	β	*p*	*b*	*SE*	β	*p*	*b*	*SE*	β	*p*
(Intercept)	-0.14	0.02	-0.26	< .001	0.14	0.03	-0.04	< .001	0.15	0.03	0.19	< .001
Accuracy	-0.03	0.01	-0.34	< .001	0.08	0.01	0.50	< .001	0.09	0.01	0.61	< .001
PSP	0.02	0.01	0.18	.136	-0.04	0.02	-0.19	.112	-0.05	0.02	-0.25	.033
ECP	0.01	0.01	0.06	.594	-0.02	0.02	-0.11	.372	> -0.01	0.02	> -0.01	.989
Error Rate	0.12	0.07	1.26	.085	0.02	0.12	0.11	.885	-0.10	0.10	-0.72	.317
Accuracy x PSP	< 0.00	0.01	-0.01	.910	> -0.01	0.02	-0.01	.940	0.01	0.02	0.03	.736
Accuracy x ECP	0.01	0.01	0.14	.160	-0.02	0.01	-0.11	.208	-0.04	0.01	-0.29	.002
PSP x ECP	< 0.00	0.01	-0.04	.671	< 0.01	0.02	0.02	.804	-0.01	0.01	-0.06	.503
Accuracy x PSP x ECP	-0.02	0.01	-0.15	.043	> -0.01	0.01	-0.01	.917	< 0.01	0.01	0.01	.932

Supplementary Table 3

Regression coefficient (b), standard error (SE), standardized regression coefficient (β), and p-value (p) for the predictors in the mixed models controlling for age

Predictor	Error rate				Bias				Sensitivity			
	b	***SE***	**β**	***p***	***b***	***SE***	**β**	***p***	***b***	***SE***	**β**	***p***
(Intercept)	0.12	0.05	0.06	.013	1.69	0.12	-0.05	< .001	0.81	0.13	0.07	< .001
PSP	-0.04	0.02	-0.23	.066	0.07	0.05	0.17	.184	-0.05	0.06	-0.10	.431
ECP	0.06	0.02	0.43	< .001	-0.11	0.04	-0.36	.004	0.08	0.04	0.23	.066
Age	< 0.01	< 0.01	0.23	.020	> -0.01	< 0.01	-0.01	.330	0.01	< 0.01	0.17	.118
PSP x ECP	-0.02	0.01	-0.10	.254	0.04	0.04	0.09	.328	-0.05	0.04	-0.13	.196

Supplementary Table 4

Regression coefficient (b), standard error (SE), standardized regression coefficient (β), and p-value (p) for the predictors in the mixed models controlling for age

Predictor	Response time				Post-response time				Post-response accuracy			
	b	***SE***	**β**	***p***	***b***	***SE***	**β**	***p***	***b***	***SE***	**β**	***p***
(Intercept)	742.85	16.2	0.10	< .001	11.51	10.12	0.04	.258	0.87	0.05	-0.07	< .001
Accuracy	22.11	7.88	0.15	.006	24.60	5.50	0.65	< .001	> -0.01	0.01	-0.01	.904
PSP	-1.19	7.78	-0.01	.879	-6.49	4.49	-0.14	.151	0.04	0.02	0.25	.033
ECP	-1.57	5.64	-0.01	.782	1.29	3.26	0.04	.694	-0.06	0.01	-0.43	< .001
Age	1.69	0.60	0.08	.006	0.04	0.38	0.01	.913	> -0.01	< 0.01	-0.22	.020
Accuracy x PSP	-1.74	11.16	-0.01	.876	-9.71	7.78	-0.20	.216	0.02	0.01	0.11	.127
Accuracy x ECP	-5.15	8.07	-0.04	.525	-11.88	5.66	-0.33	.039	-0.02	0.01	-0.13	.065
PSP x ECP	-5.49	5.53	-0.03	.324	-3.19	3.18	-0.07	.318	0.02	0.01	0.12	.157
Accuracy x PSP x ECP	0.72	7.95	< 0.01	.929	-5.88	5.50	-0.13	.288	< 0.01	0.01	0.02	.644

Supplementary Table 5

Regression coefficient (b), standard error (SE), standardized regression coefficient (β), and p-value (p) for the predictors in the mixed models on CSD-transformed data controlling for age

Predictor	Error Negativity [FCz]				Error Positivity [Cz]				Error Positivity [FCz]			
	b	***SE***	**β**	***p***	***b***	***SE***	**β**	***p***	***b***	***SE***	**β**	***p***
(Intercept)	-0.15	0.03	0.03	< .001	0.12	0.05	-0.01	.024	0.09	0.05	0.02	.056
Accuracy	-0.03	0.01	-0.34	< .001	0.08	0.01	0.50	< .001	0.09	0.01	0.61	< .001
PSP	0.02	0.01	0.14	.222	-0.04	0.02	-0.19	.104	-0.04	0.02	-0.22	.054
ECP	0.01	0.01	0.14	.210	-0.02	0.02	-0.10	.378	-0.01	0.02	-0.04	.721
Age	< 0.01	< 0.01	0.12	.192	< 0.01	< 0.01	0.04	.638	< 0.01	< 0.01	0.08	.384
Accuracy x PSP	> -0.01	0.01	-0.01	.910	> -0.01	0.02	-0.01	.940	0.01	0.02	0.03	.736
Accuracy x ECP	0.01	0.01	0.14	.160	-0.02	0.01	-0.11	.208	-0.04	0.01	-0.29	.002
PSP x ECP	-0.01	0.01	-0.05	.576	< 0.01	0.02	0.02	.789	-0.01	0.01	-0.04	.644
Accuracy x PSP x ECP	-0.02	0.01	-0.15	.043	> -0.01	0.01	-0.01	.917	< 0.01	0.01	0.01	.932

Response force and response certainty

Apart from the variables reported in the main text, we also investigated response force and response certainty. In both cases, we were able to replicate findings by Stahl et al. (2020). Response force was larger for correct responses (*M* ± *SE* = 288 ± 18.12) than for errors (198 ± 9.93), *b* ± *SE* = -87.98 ± 10.94, *t*(82.99) = -8.04, *p* < .001. Furthermore, the pooled certainty rating (1 = low certainty; 4 = high certainty) was higher for correct responses (3.84 ± 0.03) than for errors (3.66 ± 0.04), *b* = -0.20 ± 0.04, *t*(83.34) = -5.44, *p* < .001. For neither of the two variables, we found perfectionism-related variations.

Technical information on Figure 3D from the main text

Building on the Johnson-Neyman technique (Johnson & Neyman, 1936, for more see below), Figure 3D from the main text displays the *simple slopes* of the Response Accuracy factor (error vs. correct) for a large number of PSP-by-ECP combinations. These simple slopes are derived from the model equation obtained by the mixed effects model analysis of the Ne/c peak amplitude:

$$\begin{aligned} Amplitude = {} & -0.11 - 0.03 * \mathrm{Accuracy} + 0.02 * \mathrm{PSP} + 0.01 * \mathrm{ECP} - 0.01 \\ & * \mathrm{Accuracy} * \mathrm{PSP} + 0.01 * \mathrm{Accuracy} * \mathrm{ECP} - 0.01 * \mathrm{PSP} * \mathrm{ECP} \\ & - 0.02 * \mathrm{Accuracy} * \mathrm{PSP} * \mathrm{ECP} \end{aligned}$$

For example, to obtain the simple slope for PSP = -1 and ECP = -1, i.e. the slope of the factor Response Accuracy when PSP is -1 and ECP is -1, we insert these values into the equation:

$$\begin{aligned} Amplitude = {} & -0.11 - 0.03 * \mathrm{Accuracy} + 0.02 * (-1) + 0.01 * (-1) - 0.01 \\ & * \mathrm{Accuracy} * (-1) + 0.01 * \mathrm{Accuracy} * (-1) - 0.01 * (-1) * (-1) \\ & - 0.02 * \mathrm{Accuracy} * (-1) * (-1) \end{aligned}$$

Rearranging this equation yields:

$$Amplitude = -0.15 - 0.05 * \text{Accuracy}$$

Hence, the mixed effects model for the Ne/c amplitude yields a simple slope of -0.05 for the Response Accuracy factor for a PSP-ECP combination of [-1, -1]. We computed the simple slopes for a large number of PSP-ECP combinations ranging from [-3, -3] to [3, 3]. The resulting simple slopes are colour coded in Figure 3D. The black dots represent the observed PSP-ECP combination for each participant and serve to illustrate the empirical range of these variables.

Note that this approach is basically the extension of the Johnson-Neyman technique (Johnson & Neyman, 1936) to a two-dimensional space. For a two-way interaction between a moderator and a predictor, the Johnson-Neyman interval indicates the values of the moderator for which the predictor is significantly related to the DV. In our case, we have a three-way interaction between two moderators (PSP and ECP) and a predictor (Response Accuracy) and we aim at finding out for which PSP-ECP combinations, the predictor is significantly related to the DV. The border of the blue area in Figure 3D can hence be thought of as a two-dimensional Johnson-Neyman interval.

Technical information on Figure 4 from the main text

To obtain Figure 4 in the main text, we proceeded as follows: (1) We computed a mixed effects model predicting the amplitude at the FCz electrode site for a given time point (e.g. for the time point -100 ms) by response accuracy (error vs. correct), PSP, ECP, and all possible interactions. Next, (2) we computed the fitted amplitude for errors and correct responses, and for high and low values of PSP and ECP. To this end, we inserted all possible combinations of response accuracy (error vs. correct), PSP (M-SD, M+SD), and ECP (M-SD, M+SD) in the model equation obtained in (1). Finally, (3) this procedure was repeated for all time points

from -100 ms before the response to 500 ms after the response. For illustrative purposes, (4) we filtered the resulting waveforms using a 5 Hz low-pass filter. Note that the resulting waveforms are based on the mixed effects model and thus the entire sample (not four subsamples) and depict the expected (i.e. “fitted” or “predicted”) waveforms implied by the mixed effects models.

Figure 4 from the main text displays the waveforms at the FCz electrode which is the electrode site where we found significant perfectionism-related variations in the waveforms. Although we did not find such variations at the Cz electrode site (where we initially investigated the error positivity), we present the waveforms in Supplementary Figure 3.

Supplementary Figure 3

Fitted grand-average waveforms (derived from mixed effects models) at Cz for errors and correct responses for pure-EC perfectionists (upper left), mixed perfectionists (upper right), non-perfectionists (lower left), and pure-PS perfectionists (lower right). For illustrative purposes, the waveforms were low-pass filtered at 5 Hz.

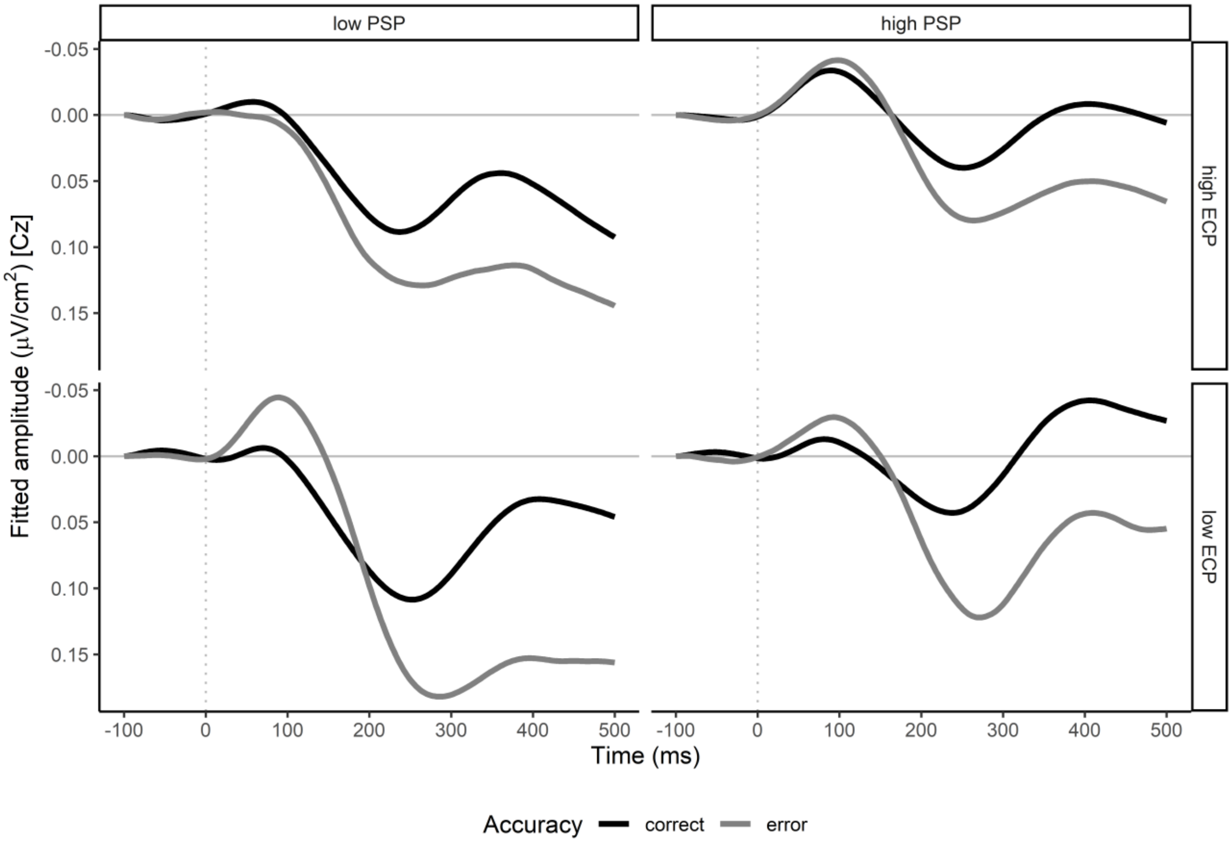

References

Arbel, Y., & Donchin, E. (2009). Parsing the componential structure of post-error ERPs: A principal component analysis of ERPs following errors. *Psychophysiology*, *46*, 1179–1189. https://doi.org/10.1111/j.1469-8986.2009.00857.x

Fischer, A. G., Klein, T. A., & Ullsperger, M. (2017). Comparing the error-related negativity across groups: The impact of error- and trial-number differences. *Psychophysiology*, *54*, 998–1009. https://doi.org/10.1111/psyp.12863

Johnson, P. O., & Neyman, J. (1936). Tests of certain linear hypotheses and their application to some educational problems. *Statistical Research Memoirs*, *1*, 57–93.

Kamarajan, C., Pandey, A. K., Chorlian, D. B., & Porjesz, B. (2015). The use of current source density as electrophysiological correlates in neuropsychiatric disorders: A review of human studies. *International Journal of Psychophysiology*, *97*, 310–322. https://doi.org/10.1016/j.ijpsycho.2014.10.013

Kayser, J., & Tenke, C. E. (2006). Principal components analysis of Laplacian waveforms as a generic method for identifying ERP generator patterns: I. Evaluation with auditory oddball tasks. *Clinical Neurophysiology*, *117*, 348–368. https://doi.org/10.1016/j.clinph.2005.08.034

Niessen, E., Fink, G. R., Hoffmann, H. E. M., Weiss, P. H., & Stahl, J. (2017). Error detection across the adult lifespan: Electrophysiological evidence for age-related deficits. *NeuroImage*, *152*, 517–529. https://doi.org/10.1016/j.neuroimage.2017.03.015

Núñez Castellar, E., Kühn, S., Fias, W., & Notebaert, W. (2010). Outcome expectancy and not accuracy determines posterror slowing: Erp support. *Cognitive, Affective, & Behavioral Neuroscience*, *10*, 270–278. https://doi.org/10.3758/CABN.10.2.270

Overhoff, H., Ko, Y. H., Feuerriegel, D., Fink, G. R., Stahl, J., Weiss, P. H., Bode, S., & Niessen, E. (2021). Neural correlates of metacognition across the adult lifespan. *Neurobiology of Aging*, *108*, 34–46. https://doi.org/10.1016/j.neurobiolaging.2021.08.001

Ruchsow, M., Spitzer, M., Grön, G., Grothe, J., & Kiefer, M. (2005). Error processing and impulsiveness in normals: evidence from event-related potentials. *Cognitive Brain Research*, *24*, 317–325. https://doi.org/10.1016/j.cogbrainres.2005.02.003

Stahl, J., Mattes, A., Hundrieser, M., Kummer, K., Mück, M., Niessen, E., Porth, E., Siswandari, Y., Wolters, P., & Dummel, S. (2020). Neural correlates of error detection during complex response selection: Introduction of a novel eight-alternative response task. *Biological Psychology*, *156*, 107969. https://doi.org/10.1016/j.biopsycho.2020.107969

Tenke, C. E., & Kayser, J. (2005). Reference-free quantification of EEG spectra: Combining current source density (CSD) and frequency principal components analysis (fPCA). *Clinical Neurophysiology: Official Journal of the International Federation of Clinical Neurophysiology*, *116*, 2826–2846. https://doi.org/10.1016/j.clinph.2005.08.007

van Veen, V., & Carter, C. S. (2002). The Timing of Action-Monitoring Processes in the Anterior Cingulate Cortex. *Journal of Cognitive Neuroscience*, *14*, 593–602. https://doi.org/10.1162/08989290260045837
